# Community of natural lactic acid bacteria and silage fermentation of corn stover and sugarcane tops in Africa

**DOI:** 10.5713/ajas.19.0348

**Published:** 2019-10-22

**Authors:** Yimin Cai, Zhumei Du, Seishi Yamasaki, Damiao Nguluve, Benedito Tinga, Felicidade Macome, Tetsuji Oya

**Affiliations:** 1 Japan International Research Center for Agriculture Sciences (JIRCAS), Tsukuba, Ibaraki 305-8686, Japan; 2 College of Grassland Science and Technology, China Agricultural University, Beijing 100193, China; 3 Agricultural Research Institute of Mozambique, Matola 999068, Mozambique

**Keywords:** Corn Stover, Lactic Acid Bacteria, Microbial Population, Silage Fermentation, Sugarcane Top

## Abstract

**Objective:**

To effectively utilize crop by-product resources to address the shortage of animal feed during the dry season in Africa, the community of natural lactic acid bacteria (LAB) of corn stover and sugarcane tops and fermentation characteristics of silage were studied in Mozambique.

**Methods:**

Corn stover and sugarcane tops were obtained from agricultural field in Mozambique. Silage was prepared with LAB inoculant and cellulase enzyme and their fermentation quality and microbial population were analyzed.

**Results:**

Aerobic bacteria were the dominant population with 10^7^ colony-forming unit/g of fresh matter in both crops prior to ensiling, while 10^4^ to 10^7^ LAB became the dominant bacteria during ensiling. *Lactobacillus plantarum* was more than 76.30% of total isolates which dominated silage fermentation in the LAB-treated sugarcane top silages or all corn stover silages. Fresh corn stover and sugarcane tops contain 65.05% to 76.10% neutral detergent fiber (NDF) and 6.52% to 6.77% crude protein (CP) on a dry matter basis, and these nutrients did not change greatly during ensiling. Corn stover exhibits higher LAB counts and water-soluble carbohydrates content than sugarcane top, which are naturally suited for ensiling. Meanwhile, sugarcane tops require LAB or cellulase additives for high quality of silage making.

**Conclusion:**

This study confirms that both crop by-products contain certain nutrients of CP and NDF that could be well-preserved in silage, and that they are potential roughage resources that could cover livestock feed shortages during the dry season in Africa.

## INTRODUCTION

The major constraint for cows in the tropics is shortage of feed in terms of quality and quantity, especially in the dry season [1]. The main roughage sources for cows in Africa are native grasses and agricultural by-products. When cows are not fed such quality roughage, decreased milk and meat production results [2]. In recent years, improved technologies for producing forage crops, grasses, and crop by-products have been investigated, and their adaptability to various conditions, nutritive value, and contribution to animal productivity explored [3]. Several factors can influence crops nutrition, such as plant genotype, sowing density, harvest season, irrigation and fertilization. Generally, tropical crops can only be grown during the rainy season and the bulk of by-product production occurs after harvest. Crop by-products should be preserved to ensure a continuous supply of feed for ruminants during the dry season. Improved silage preparation and storage are the most effective techniques for ensuring that the animal production system can cope with animal feed shortages in the tropics [1].

Corn (*Zea mays* L.) and sugarcane (*Saccharum officinarum* L.) are important crops for food and sugar production in Africa, and they were produced approximately 1.45 million tons and 2.76 million tons annually, respectively in Mozambique [4]. When corn is harvested for grain, more than 40% dry matter (DM) of the corn plant, including the leaves, stalks, husks, and cobs, are discarded in the field [5]. Sugarcane top as residue represent 15% to 25% of the aerial part of the plant [6]. Therefore, corn stover and sugarcane tops are among the main agricultural by-products produced in Africa, but they are generally discarded in the field, where they are often burned and used as fertilizer [7].

The by-products of both corn and sugarcane could be used for livestock feed, as they are cheap and abundant in the tropics, where other green fodder is unavailable. The preservation of forage crops, as silage, depends on the production of sufficient acid to inhibit the activity of contaminating microbes under anaerobic conditions [8]. Usually, it is difficult to make tropical forage crops and grasses of high fermentation quality due to their high moisture and low water-soluble carbohydrates (WSC) contents [2]. Better technologies aimed at creating good-quality animal feed, by providing long-term storage of silage from crop by-products, need to be developed. The improvement of ensiling techniques through use of biological additives, such as lactic acid bacteria (LAB) inoculants and cellulose, is widely anticipated. It is well-accepted that these silage additives could improve silage preservation efficiency and thereby enhance cattle performance [9]. In general, cellulase improves fibre degradation by increasing the WSC as a substrate for LAB, which then produce lactic acid. As a result, the pH decreases, preserving the forage crops.

Ensiling methods for creating good-quality silage and long-term storage are not available year-round in the tropics [1]. However, there is limited information available on epiphytic LAB characteristics and silage fermentation in Africa. In the present study, we identified and characterised LAB isolated from fresh corn stover and sugarcane tops in Mozambique, and then determined their ensiling characteristics. To improve fermentation quality, the silages were prepared with microbial additives, including LAB inoculants and cellulase enzymes, which are likely to play an important role in improving silage fermentation.

## MATERIALS AND METHODS

### Ensiling materials and silage preparation

Corn stover and sugarcane tops were obtained from agricultural field in a corn-production area and in an industrial sugar-production region in Maputo, Mozambique on September 2018, respectively. After harvest, fresh corn stover and sugarcane tops were immediately cut into 1 to 2 cm lengths by a chopper (92-2S, Sida Agri-Machine Co., Ltd, Luoyang, China), and approximately 8 kg were packed into 20 L polyethylene drum (Ka-Kosher Co., Ltd, Sinaloa, Mexico) silos. The silos were kept at an ambient temperature (25°C to 38°C) and opened after 60 d of ensiling for analysis of fermentation quality and microbial analysis [10]. The experiment was designed as a 2×4 factorial arrangement in a completely randomized design (crop by-products × additive treatments) with triple replicates per treatment. The commercial LAB inoculant FG (*Lactobacillus plantarum*, Snow Brand Seed Co., Ltd, Sapporo, Japan) and Acremonium cellulase (*Acremonium cellulase*, Meiji Seika Pharma Co., Ltd, Tokyo, Japan) were used as silage additives based on the guidelines of a commercial manufacturer. Inoculant strain originally isolated from forage crop that could produce more lactic acid in the silage environment. Cellulase is produced from *Acremonium cellulolyticus*, the main compositions are glucanase and pectinase, carboxymethyl cellulase activity is 7,350 U/g. The LAB were inoculated at 5 mg/kg as 1.0×10^5^ colony-forming unit (cfu)/g on a fresh matter (FM) basis. Cellulase was added at 10 mg blended with 20 mL H_2_O per kg of FM. Silage treatments were designed as control, LAB, cellulase, and LAB+cellulase. The LAB and cellulase were diluted with deionized water, and the additive solution was sprayed using an electronic sprayer (SSP-5H, fujiwara Sangyo Co., Ltd, Miki, Japan) for addition of experiment treatments. The same amount of deionized water was sprayed on the control treatment.

### Microbial analysis

The counts of microorganisms in the crop by-products or silages were measured by the plate count method [11]. Samples (10 g) were blended with 90 ml sterilized water and serially diluted 10^−1^ to 10^−8^ in sterilized water. The numbers of LAB were measured on Lactobacilli MRS (de Man, Rogosa and Sharpe) agar (Difco Laboratories, Detroit, MI, USA) incubated at 30°C for 48 h under anaerobic conditions (Anaerobic Pack Rectangular Jar; 2.5 liters, Mitsubushi Gas Chemical Company INC, Tokyo, Japan). For isolation of LAB, more than 20 strains on MRS agar medium were picked randomly from each sample, and a total of 97 isolates were collected, of which 75 isolates were considered to be LAB, as determined by the Gram-stain appearance, catalase test and lactic acid productivity [11]. Aerobic bacteria were counted on Nutrient agar (Nissui-Seiyaku Co., Ltd, Tokyo, Japan) incubated for 48 h at 30°C under aerobic conditions. Coliform bacteria were counted on Blue Light agar (Nissui-Seiyaku, Japan) incubated at 30°C for 48 h; mold and yeast were counted on Potato Dextrose agar (Nissui-Seiyaku, Japan) incubated for 48 to 72 h at 30°C. Yeasts were distinguished from mold and bacteria by colony appearance and observation of cell morphology. Colonies were counted as viable numbers of microorganisms in cfu/g of FM. For LAB identification, each colony of LAB was purified twice by streaking on a MRS agar plate. The pure cultures were grown on MRS agar at 30°C for 24 h, resuspended in a solution of nutrient broth (Difco, USA) and dimethyl sulfoxide at a ratio of 9:1, and stored as stock cultures in a deep freezer (MDF-U384, Sanyo Electric Co., Ltd, Osaka, Japan) at −80°C until further examination.

Gram stain and morphological characteristics of LAB were determined after 24 h of incubation on MRS agar, and their catalase activity and gas production from glucose were determined as described by Kozaki et al [11]. Growth at different temperatures was detected in MRS broth after incubation at 5°C and 10°C for 10 d, and at 40°C, 45°C, and 50°C for 7 d. Growth at pH 3.0 to 7.0 was observed in MRS broth after incubation at 30°C for 7 d. Carbohydrate assimilation and fermentation of 49 different compounds with one control were identified on AP 50 CH strips. These strains were divided into five groups (A to E) according to morphological and biochemical characters and 16S rRNA sequence analysis, and the representative strains of each group were selected by their different fermentation patterns of AP 50 CH.

### 16S rRNA gene sequence analysis

Cells of representative strains grown for 8 h in MRS broth at 30°C were used for DNA extraction and purification [12]. The 16S rDNA sequence coding region was amplified by polymerase chain reaction (PCR) and performed in a PCR ThermalCycler (GenAmp PCR System 9700; PE Applied Biosystems, Foster City, CA, USA) and reagents from a Takara *Taq* PCR Kit (Takara Shuzo Co., Ltd, Otsu, Japan). Sequencing was performed twice on both strands by the dideoxy method using a PRISM BigDye Terminator Cycle Sequence Ready Reaction Kit (Applied Biosystems, USA) in combination with an Applied Biosystems model 310 A automated sequencing system. Searching 16S rDNA sequence similarity was performed at GenBank data library by using the BLAST program. Then the sequence information was introduced into the CLUSTAL W software program (Hitachi Software Engineering Co., Ltd, Tokyo, Japan) for assembly and alignment [13]. The 16S rDNA sequences of isolates were compared with sequences from the strains of LAB held in the GenBank. Nucleotide substitution rates were calculated, and phylogenetic trees were constructed by the neighbor-joining method. *Bacillus subtilis* NCDO 1769 was used as an outgroup organism [11]. The topology of trees was evaluated by bootstrap analysis of the sequence data with CLUSTAL W software based on 100 random resamplings [13].

### Chemical analysis

Pre-ensiled corn stover and sugarcane tops, and their silage samples were dried in a forced air oven at 70°C for 48 h, and ground to pass a 1 mm mesh screen (FW 100, Taisite Instrument Co., Ltd, Tianjin, China) for chemical composition analyses. The data of chemical composition on DM basis were corrected for residual moisture after 3 h at 105°C. The DM, ash, crude protein (CP) and ether extract (EE) were analyzed by the methods 934.01, 942.05, 976.05, and 920.39 of AOAC [14], respectively. The organic matter (OM) content was calculated as the weight loss upon ashing. The neutral detergent fiber (NDF) and acid detergent fiber (ADF) were obtained according to the method of Van Soest et al [15] with an ANKOM A200i fiber analyzer (ANKOM Technology, Macedon, NY, USA) and were expressed exclusive of residual ash. The acid detergent lignin (ADL) analysis was subsequently performed following the procedure of Van Soest et al [15]. The WSC was determined by Anthron method [16]. Lactate buffer capacity (LBC) was measured by titrating with 0.1 M HCl to reduce pH from initial pH to pH 3.0 and then titrated to pH 6.0 with 0.1 M NaOH as described by McDonald et al [17]. Degradable intake protein (DIP) was analyzed by the method of Roe et al [18]. Undegraded intake protein (UIP) was calculated as described by Licitra et al [19]. Soluble intake protein (SIP) was analyzed by the method of Licitra et al [19] in omitting sodium azide. Binding protein (BP) and neutral detergent insoluble protein (NDIP), the CP contents of sequential NDF residue were determined with the method of Licitra et al [19]. Pre-ensiled material and samples preparation for macro mineral analysis was carried out as described by Khan et al [20]. The concentration of calcium (Ca), phosphorous (P), magnesium (Mg), and potassium (K) was measured using an atomic absorption spectrophotometer (PerkinElmer, LAMBDA 1050, Shelton, CT, USA).

### Fermentation analysis

The fermentation products of silage were analyzed by using cold-water extract, a 10 g wet silage sample was homogenized with 90 mL of deionized water and kept in a refrigerator at 4°C for 24 h as described by Cai [10]. Then, the material was filtered, and the filtrate was used to measure pH, ammonia-N, and organic acids. The pH was measured using a glass electrode pH meter (Starter 100/B, OHAUS, Shanghai, China), the ammonia-N content was analyzed by using steam distillation of the filtrates [10], the concentration of organic acid including lactic acid, acetic acid, propionic acid and butyric acid were measured by high-performance liquid chromatography (Sodex RS Pak KC-811column; Showa Denko K.K., Kawasaki, Japan; DAD detector: 210 nm, SPD-20A, Shimadzu Co. Ltd., Kyoto, Japan; eluent: 3 mm HClO4, 1.0 mL/min; temperature: 40°C) methods as described by Cai [10].

### Statistical analysis

Data on the microorganism population, chemical composition and fermentation quality after 60 d of ensiling were analyzed with a completely randomized design with a 2×4 (crops [C] × additives [A]) factorial treatment structure. The two ways analysis of variance (ANOVA) procedure of SAS version 9.1 (SAS Institute, Cary, NC, USA) was used for the analysis and the statistical model is as follows:

$$Y_{\text{ijk}}=\mu +\alpha_{\text{i}}+\beta_{\text{j}}+\alpha \beta_{\text{ij}}+{ɛ}_{\text{ijk}}$$

where Y_ijk_ = observation; μ = overall mean, α_i_ = crops effect (i = corn stover and sugarcane top), β_j_ = additives effect (j = 1 to 4), αβ_ij_ = crops×additives effect, and ɛ_ijk_ = error. The mean values were compared by Tukey’s test [21].

### Accession numbers

The nucleotide sequences for the 16S rDNA described in this report were deposited with GenBank under accession numbers LC434015, LC434016, LC434017, LC434018, and LC434019 for the strains MB1, MB14, MB6, MB52, and MB38, respectively.

## RESULTS

The chemical, protein and macro mineral composition of corn stover and sugarcane tops are shown in Table 1. The DM content of the fresh corn stover was 16.97% higher (p<0.05) than that in fresh sugarcane tops. The CP and EE contents of both crop by-products did not show marked differences. The OM, NDF, ADF, ADL, and LBC contents of corn stover were lower (p<0.05) than those of respective values in sugarcane tops. The WSC content of corn stover was 2.53% of DM higher (p<0.05) than sugarcane tops. Regarding the protein composition, the DIP and SIP contents were higher (p<0.05), and the UIP and NDIP contents lower (p<0.05), in the corn stover than in sugarcane tops. The BP contents were similar in both crops. Regarding the macro minerals, the K contents were similar in both crops, while the Ca, P, and Mg contents were higher (p<0.05) in the corn stover.

The microbial population of the corn stover and sugarcane tops before ensiling are shown in Table 2. Aerobic bacteria dominated both crops with similar levels of 10^7^ cfu/g in FM. The corn stover contained 10^7^ coliform bacteria, 10^6^ yeasts, and 10^4^ molds cfu/g in FM. The microbial counts for all three microorganisms were lower in the sugarcane tops than corn stover. The natural LAB, including the genera *Lactobacillus*, *Weissella*, *Lactococcus*, and *Pediococcus*, were present at 10^3^ to 10^6^ cfu/g of FM in both crops.

The characteristics of LAB of corn stover and sugarcane tops and their silages are shown in Table 3. Representative strains MB1, MB6, MB14, MB38, and MB52 were isolated from both crops or their silages. A total of 97 strains were isolated from MRS agar plates. Of those, 22 strains did not exhibit characteristics of LAB, namely Gram-negative, catalase-positive, and unable to produce lactic acid in MRS broth. The other 75 strains were considered to be LAB, they were Gram-positive and catalase-negative rods or cocci. Those strains are able to form D(−) isomer, L(+) isomer, or approximately equal quantities of D(−) and L(+)-lactic acid, and did or did not produce gas from glucose.

**Figure 1:**
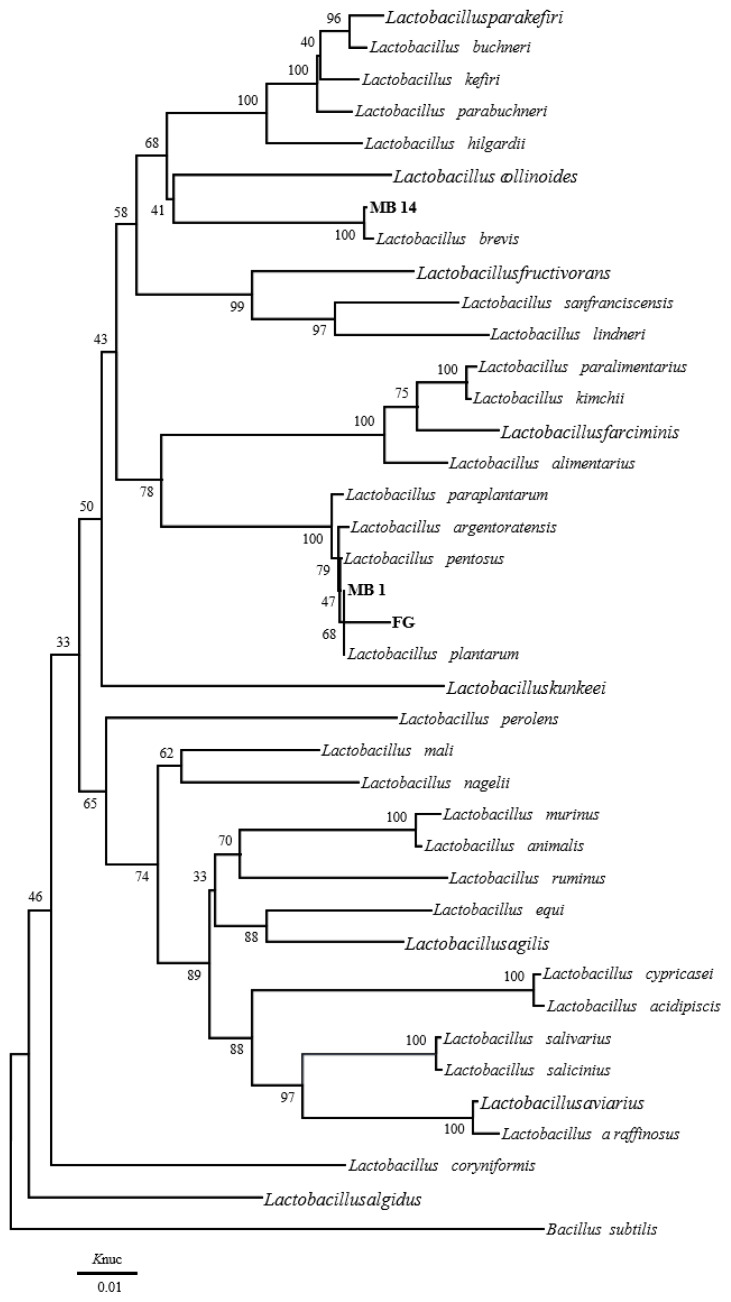
Phylogenetic tree showing the relationship between the 16S rDNA sequences of rod-shaped LAB strains obtained in this study. Numbers at nodes are bootstrap values based on a neighbor-joining bootstrap analysis with 1,000 replications. *Bacillus subtilis* is used as the outgroup. The bar indicates 1% sequence divergence. *Knuc*, nucleotide substitution rates; LAB, lactic acid bacteria.

**Figure 2:**
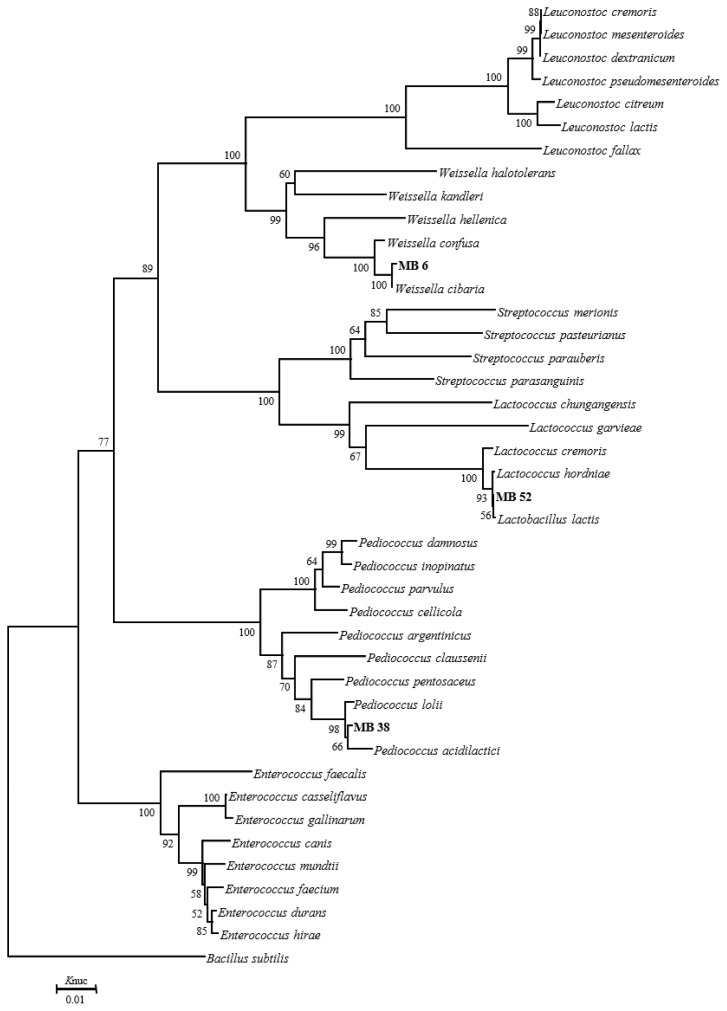
Phylogenetic tree showing the relationship between the 16S rDNA sequences of cocci-shaped LAB strains obtained in this study. Numbers at nodes are bootstrap values based on a neighbor-joining bootstrap analysis with 1,000 replications. *Baccillus subtilis* is used as the outgroup. The bar indicates 1% sequence divergence. *Knuc*, nucleotide substitution rates; LAB, lactic acid bacteria.

The phylogenetic trees of 16S rRNA gene sequence for rod-shaped and cocci-shaped LAB strains obtained in this study are shown in Figure 1, 2, respectively. More than 1,500 bases of 16S rRNA of these strains were determined. Following phylogenetic analysis, the rod-shaped strains MB1, FG, and MB14 were placed in the cluster making up the genus *Lactobacillus*. Strains MB14 were clearly assigned to the *Lactobacillus brevis*. While strains MB1 and FG grouped on the phylogenetic tree together with *L. plantarum*, including species, *L. pentosus*, *L. argentoratensis*, and *L. paraplantarum*, and in a 100% bootstrap cluster. Furthermore, strains MB1 and FG appeared to be equally linked to *L. plantarum*, these strains were phylogenetically associated with *L. plantarum*. Cocci-shaped strains MB6, MB38, and MB52 were placed in the cluster making up the genus *Weissella*, *Lactococcus*, and *Pediococcus*, respectively. These type strains of *Weissella cibaria*, *Lactococcus lactis*, and *Pediococcus acidilactici* were the species most closely to the strains MB6, MB38, and MB52 in the phylogenetic tree, and they showed the sequence similarity value more than 99.80% with each strain. Based on the morphological and biochemical characteristics, and 16S rRNA gene sequence analysis, these isolates were identified as *Lactobacillus plantarum*, *Lactobacillus brevis*, *Weissella cibaria*, *Lactococcus lactis*, and *Pediococcus acidilactici*.

**Figure 3:**
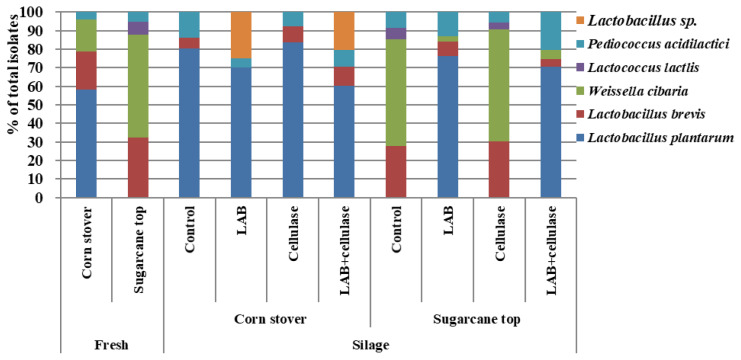
Community of lactic acid bacteria of corn stover, sugarcane top and silage. LAB, lactic acid bacteria inoculant FG; Cellulase, *Acremonium* cellulase.

The LAB community of corn stover and sugarcane tops and their silages are shown in Figure 3. Microbial diversity of LAB was observed in corn stover, where *Lactobacillus plantarum* (58.40%; percentage of total isolates), *Lactobacillus brevis* (20.60%), *Weissella cibaria* (17.00%), and *Pediococcus acidilactici* (4.00%) were the predominant species. In the sugarcane tops, the LAB microbial communities included *Lactobacillus brevis* (32.20%), *Weissella cibaria* (55.60%), *Lactococcus latcis* (7.20%), and *Pediococcus acidilactici* (5.00%). *Lactobacillus plantarum*, the dominant bacterium in the corn stover, was not detected in the sugarcane tops. *Weissela cibaria* was the dominant LAB in sugarcane tops. *Lactobacillus brevis* and *Pediococcus acidilactici* were abundant in both crops. After 60 d of fermentation, *L. plantarum* was the dominant LAB species (76.30% to 83.60% of total isolates) in the LAB− and LAB+celllulase-treated sugarcane tops, and all corn stover silages, while *Weissella cibaria* (57.40% to 60.50%) was the dominant species in control and cellulase-treated sugarcane top silages.

The chemical composition of corn stover and sugarcane top silages are shown in Table 4. After 60 d of ensiling, the CP contents of the corn stover and sugarcane tops silages were similar, ranging from 5.99% to 6.57% and 6.00% to 6.28% of DM, respectively. The contents of OM and EE in by-product silages did not show marked differences from the control, LAB, cellulase or combined LAB and cellulase treatments. However, the NDF and ADF contents of the cellulase-treated silages were lower (p<0.05) than those of control and LAB-treated silages. Compared to corn stover silages, the OM, EE, NDF, ADF, and ADL contents of sugarcane tops silages were higher (p<0.05). Crops (C) influenced OM, EE, NDF, ADF, and ADL contents (p = 0.0073 to 0.0164 or p<0.0001), but the CP (p = 0.3059) did not. The additives (A) influenced (p≤ 0.0001) NDF and ADF contents, while the other chemical composition did not (p = 0.2580 to 0.6179). The interaction between C and A (C×A) influenced (p = 0.0344 to 0.0445) NDF and ADL contents, but not OM, CP, EE, or ADF (p = 0.2932 to 0.9777).

The fermentation quality of the corn stover and sugarcane top silages after 60 d of fermentation is shown in Table 5. All of the corn stover silages were well-preserved, with low pH values (<3.97) and ammonia-N content (<0.77% g/kg of DM), and high lactic acid content (>4.14% of DM). The fermentation quality of the control, LAB−, cellulase−, and LAB+ cellulase-treated corn stover silages did not display marked differences. In contrast, the fermentation quality of sugarcane top silages varied markedly. After 60 d of fermentation, the control silage was of poor quality, with low lactic acid content (0.61% of DM) and a relatively high pH value (4.71). However, the LAB−, cellulase−, and LAB+cellulase-treated silages had similar good fermentation patterns, with higher (p<0.05) lactic acid contents and lower (p<0.05) pH than those of the control silage. The DM and lactic acid content were higher (p<0.05), and the pH and butyric acid content lower (p<0.05), in corn stover silages compared with sugarcane top silages. The acetic acid, propionic acid, and ammonia-N contents were similar between the corn stover and sugarcane top silages. The C influenced (p = 0.0005 to 0.0088 or p<0.0001) silage DM, pH, lactic acid, butyric acid and ammonia-N contents, while the A and A×C influenced pH (p = 0.0012 to 0.0024), lactic acid (p = 0.0072 to 0.0073), acetic acid (p = 0.0008 to 0.0160), butyric acid (p = 0.0005 to 0.0008) and ammonia-N (p = 0.0071 to 0.0111). The A also influenced (p = 0.0434) the propionic acid content.

The microbial populations of corn stover and sugarcane top silages after 60 d of fermentation are shown in Table 6. For the corn stover silages, the microbial populations were similar among all treatments; LAB (10^6^ cfu/g of FM) was the dominant species. Additionally, the aerobic bacteria count was 10^4^ to 10^5^ cfu/g, and that of yeasts was 10^5^ cfu/g of FM. Meanwhile, coliform bacteria and molds were below detectable levels (<10^2^ cfu/g of FM). For the sugarcane top silages, 10^4^ to 10^7^ LAB, 10^3^ to 10^5^ aerobic bacteria, and 10^3^ to 10^5^ yeasts counts were presented in the silages. In the LAB−, cellulase− and LAB+cellulase-treated silages, the LAB count was significantly (p<0.05) higher than that of the control, while that of aerobic bacteria was significantly (p<0.05) lower. Similar to the corn stover silages, the counts of coliform bacteria and molds in all sugarcane top silages were below detectable levels (<10^2^ cfu/g of FM). The C, A, and C×A influenced (p = 0.0002 to 0.0213 or p<0.0001) the LAB, aerobic bacteria, and yeast counts.

## DISCUSSION

In general, the chemical compositions of tropical grasses and forage crops were different, especially with regard to DM, CP, NDF, and WSC contents. Some tropical grasses are less amenable to producing good-quality silage due to their low DM, high LBC, and low WSC contents [22]. The density is also an important factor for silage fermentation. In this study, the weight of corn and sugar cane silage packed into silo is 7.85 to 8.38 kg. Although the moisture of the two kinds of silages is different, there is no large difference in density between the two silages. This may be because the top of the sugar cane is rich in leaf parts, while the corn stover is rich in stem parts. The CP and EE contents of the by-products of both crop types were at similar levels, but the corn stover exhibited more suitable ensiling characteristics, such as lower moisture content and LBC, and higher WSC content. In addition, the DIP, SIP, UIP, BP, NDIP, Ca, P, and Mg contents in the corn stover were much higher than those in sugarcane tops, suggesting that corn stover contains high amounts of digestible feed ingredients that could contribute to livestock feed.

To comprehensively understand the microbial populations in crop by-products and their silages, we investigated the abundance of four kinds of microbes: LAB, aerobic bacteria, molds, and yeasts. Generally, farm silage is based on natural lactic acid fermentation, and epiphytic LAB from forage transform the WSC into organic acids during the ensiling process. As a result, the pH is reduced and the forage preserved [23]. Therefore, the abundance, taxonomy, and characteristics of epiphytic LAB have become significant factors for predicting the adequacy of silage fermentation and determining whether to apply bacterial inoculants to silage materials [23]. When LAB are present in low numbers in forage crops or grasses, they will fail to produce sufficient lactic acid during fermentation to reduce the pH and inhibit the growth of clostridia; therefore, the resulting silage will be of poor quality. In this study, the microbial population between the two crops is different. The reason for this is unclear. Perhaps the cultivation environment and their chemical composition of both crops, especially moisture and WSC contents may influence the distribution of native microorganisms [24]. Relatively high numbers of LAB (>10^5^ cfu/g of FM) were present in the corn stover in this study. In particular, *Lactobacilli* were dominant within the LAB populations. In this case, it would not be necessary to use bacterial inoculants to control contaminating microbes during silage fermentation (Table 1). However, very low numbers of LAB (<10^3^ cfu/g of FM) were observed in sugarcane tops, and *Weissella* were the dominant species in those populations; this suggests that it is necessary to use LAB inoculant to improve silage fermentation.

The isolates from this study were Gram-positive and catalase-negative rods or cocci that produced lactic acid from glucose. These properties suggest that these strains belong to the LAB species. The representative strains exhibited differences in terms of gas production from glucose, yielding approximately equal quantities of L(+) and D(−)-lactic acid, but they could not be identified down to the species level on the basis of these phenotypic characteristics. The identification and genetic interrelationships of the LAB, including new species isolated from silage, have been studied extensively in 16S rRNA gene sequence and DNA-DNA hybridisation experiments [23]. Recent results indicate that the LAB genera *Lactobacillus*, *Pediococcus*, *Leuconostoc*, *Weissella*, and *Lactococcus* exhibit a high degree of sequence similarity to one another and form a phylogenetically coherent group that is separate from other bacteria [1]. In the present study, isolated strains were of the genera *Lactobacillus* (two strains), *Weissella*, *Lactococcus*, and *Pediococcus* based on the phylogenetic analysis, thus confirming that these strains belong to these LAB genera. The representative strains MB1, MB6, MB14, MB38, and MB52 are the species most closely related to type strains of *Lactobacillus plantarum*, *Lactobacillus brevis*, *Weissella cibaria*, *Lactococcus lactis*, and *Pediococcus acidilactici*, respectively. The 16S rDNA sequence similarity of these strains was more than 99.80% to each other, and less than 98.00% to other type strains. The AP 50 CH data also supported these results, and these strains and their type strains had similar fermentation patterns. To our knowledge, this is also the first report of natural LAB community on Africa silage.

Certain lactic acid-producing cocci create an aerobic environment suitable for the development of lactobacilli only during the early stages of the silage fermentation process. In contrast, *Lactobacilli* (rods) are important promoters of lactic acid fermentation for longer periods [23]. Many studies have reported that homofermentative lactobacilli, such as *Lactobacillus casei* and *Lactobacillus plantarum*, promote lactic acid fermentation and improve silage quality [1]. In the present study, all corn stover silages were well-preserved, with high lactic acid contents and low pH values. The factors used to assess fermentation quality included the chemical composition of the corn stover material and the physiological properties of epiphytic LAB. The corn stover had a relatively high WSC content and low LBC, and a high number of epiphytic LAB (>10^5^ cfu/g of FM). *Lactobacillus plantarum* are the dominant species naturally distributed in corn stover [25]; this species is a homofermentative type of LAB which grows well under low pH conditions and produces more lactic acid than other strains [26]. During ensiling, lactobacilli can use sugars to increase the production of lactic acid, thereby reducing the pH and inhibiting the growth of harmful bacteria, in turn resulting in good-quality silage. On the other hand, the control silage of the sugarcane top was of poor quality, while the microbial additive-treated silages were of good quality. The parameters for assessing fermentation quality include the chemical composition of the crop materials and the physiological properties of the epiphytic LAB. The sugarcane tops had a relatively low WSC content (<7.85% of DM), and the LAB could not ferment sufficient sugar to produce lactic acid (Table 4). Furthermore, the pH value of this silage did not decrease below 4.0, such that clostridia was present via butyric acid fermentation and ammonia-N production. However, when the sugarcane top silage was treated with LAB or cellulose, fermentation quality was higher. The cellulase addition could improve fibre degradation to increase WSC as a substrate for LAB, to produce lactic acid and thereby improve fermentation quality. *Acremonium* cellulase used in this study contain a strong activity of glucanase and pectinase, it can play an important role in the silage fermentation process [27]. It is suggested that a decrease in fibre content, including NDF and ADF, in sugarcane top silages underlies this phenomenon. Thus, if crops such as corn have sufficient sugar content, even without the addition of LAB, it would be possible to make a good-quality silage. Therefore, LAB and cellulase might influence the fermentation quality of silage. When the crop by-product contains an insufficient amount of LAB and WSC, it is necessary to add them for silage fermentation.

The present study mainly focused on silage preparation and ensiling characteristics analysis. The future experiment will be required to study the digestibility of these crop by-product silages for sheep or cows. Now, the silage or total mixed ration prepared by using local available resources and their evaluation on the digestibility and milk production of dairy cattle is under process. In Africa, agricultural residues from crop production have increased rapidly in recent years [28], in turn increasing the need for efficient use of crop by-products for economic and environmental reasons. Silage prepared with local crop by-products could be a very effective fermentation technology.

## CONCLUSION

This study suggests that corn stover contained effective LAB species for natural silage fermentation; meanwhile, sugarcane tops could yield good silage with LAB inoculant. Based on the silage fermentation and chemical composition analyses, corn stover and sugarcane tops contain an abundance of nutrients for livestock. Fresh corn stover exhibits good ensiling characteristics and high levels of LAB, which are naturally suited for ensiling and fermentation. Meanwhile, sugarcane tops require LAB or cellulase additives for silage production. This study suggests that by-products of both crops, as silage, are well-suited for preservation of forage crops and could serve as roughage sources to cover animal feed shortages during the dry season in Africa.

## IMPLICATIONS

The present study reports the community of natural LAB and silage fermentation of corn stover and sugarcane tops, focus on addressing feed shortage during dry season in Africa. Corn stover and sugarcane tops contain an abundance of nutrients for animal. The LAB, especially *Lactobacillus plantarum* became the dominant bacteria affect silage fermentation after ensiling. Fresh corn stover exhibits good ensiling characteristics and high levels of LAB, which are naturally suited for ensiling and fermentation. Meanwhile, fresh sugarcane tops require LAB or cellulase additives for silage preparation. Based on the bacterial community and silage fermentation analyses, the crop by-products can be well-preserved as silage and have great potential as a feed source for livestock, to cover feed shortages during the dry season in Africa. Silage prepared with local available crop by-product resources could be a very effective fermentation technology for animal production.

## Figures and Tables

**Table 1:** Chemical, protein and macro mineral composition of corn stover and sugarcane tops

Items	Corn stover	Sugarcane top	SEM	p-value
Chemical composition				
DM (%)	42.65±1.67^a^	25.68±2.08^b^	1.09	0.0004
OM (% of DM)	84.22±2.55^b^	94.65±0.87^a^	1.10	0.0026
CP (% of DM)	6.52±0.20	6.77±0.78	0.33	0.6165
EE (% of DM)	1.57±0.60	1.80±0.28	0.30	0.6319
NDF (% of DM)	65.05±0.50^b^	76.10±2.01^a^	0.85	0.0008
ADF (% of DM)	35.08±3.62^b^	42.46±1.04^a^	1.54	0.0274
ADL (% of DM)	3.49±0.54^b^	5.11±0.36^a^	0.26	0.0119
WSC (% of DM)	10.38±0.40^a^	7.85±0.39^b^	0.23	0.0014
LBC (meq/ kg of DM)	539.75±21.23^b^	1,772.40±183.30^a^	75.33	0.0003
Protein composition (% of CP)				
DIP	69.34±1.51^a^	61.36±2.08^b^	1.05	0.0051
UIP	30.66±1.75^b^	38.64±1.40^a^	0.92	0.0034
SIP	39.31±1.34^a^	24.66±0.69^b^	0.61	<0.0001
BP	25.90±4.03	24.08±4.07	2.34	0.6115
NDIP	39.67±3.98^b^	50.59±1.22^a^	1.70	0.0105
Macro mineral (mg/kg)				
Ca	0.35±0.01^a^	0.27±0.04^b^	0.01	0.0177
P	0.20±0.01^a^	0.14±0.01^b^	0.01	0.0031
Mg	0.21±0.01^a^	0.18±0.01^b^	0.01	0.0161

SEM, standard error of the mean; DM, dry matter; OM, organic matter; CP, crude protein; EE, ether extract; NDF, neutral detergent fiber; ADF, acid detergent fiber; ADL, acid detergent lignin; WSC, water soluble carbohydrates; LBC, lactate buffer capacity; DIP, degradable intake protein; UIP, undegradable intake protein; SIP, soluble intake protein; BP, binding protein; NDIP, neutral detergent insoluble protein; Ca, calcium; P, phosphorous; Mg, magnesium; K, potassium.

a,b Means±standard deviation within rows with different superscript letters differ significantly from each other (p<0.05).

**Table 2:** Microbial population of corn stover and sugarcane tops before ensiling

Items	Lactic acid bacteria	*Lactobacilli*	*Weissella*	*Lactococci*	*Pediococci*	Aerobic bacteria	Coliform bacteria	Yeasts	Molds

	------------------------------------------------------------------ log 10 cfu/g of FM ----------------------------------------------------------------------								
Corn stover	5.73	5.70	3.18	3.75	ND	7.89	7.72	6.41	4.12
Sugarcane top	6.56	3.08	6.54	ND	3.87	7.20	5.26	4.27	3.79

Data are means of three samples.

cfu, colony-forming unit; FM, fresh matter; ND, not detected.

**Table 3:** Characteristics of lactic acid bacteria isolated from corn stover, sugarcane tops and inoculant

Characteristic	*Lactobacillus plantarum*	*Lactobacillus brevis*	*Weissella cibaria*	*Lactococcus lactlis*	*Pediococcus acidilactici*	*Lactobacillus plantarum*
Representative strain	MB1	MB14	MB6	MB52	MB38	FG
Isolated source	Corn stover	Sugarcane tops	Corn stover	Sugarcane tops	Corn stover	Inoculant
Shape	Rod	Rod	Cocci	Cocci	Cocci	Rod
Gram stain	+	+	+	+	+	+
Catalase	−	−	−	−	−	−
Gas from glucose	−	+	+	−	−	−
Lactate production in MRS broth (%)	1.96	1.31	0.67	0.45	0.92	0.92
Final pH in MRS broth	3.50	3.85	4.40	4.60	4.20	4.20
Fermentation type	Homo	Hetero	Hetero	Homo	Homo	Homo
Optical form of lactate	DL	DL	D(−)	L(+)	DL	DL
Growth at pH						
3.0	−	−	−	−	−	−
3.5	+	−	−	−	−	−
4.0	+	+	w	−	−	−
4.5	+	+	+	w	+	+
5.0	+	+	+	+	+	+
Carbohydrate fermentation patterns						
L-Arabinose	+	+	w	−	+	+
Ribose	+	+	−	+	+	+
D-Xylose	−	+	w	+	+	−
Galactose	+	−	−	+	+	+
D-Mannose	+	−	+	+	+	+
Mannitol	+	−	−	+	−	+
α Methyl-D-mannoside	+	−	−	−	−	+
Amygdaline	+	−	+	+	+	+
16S rDNA similarity with each type strain (%)	100.00	99.80	99.90	99.80	99.90	99.90

+, positive; −, negative; w, weakly positive.

Carbohydrate fermentation pattern was tested by AP 50 CH; the sequence similarity of 16S rDNA of isolates were compared with sequences from each type strains of LAB held in the GenBank.

**Table 4:** Chemical composition of corn stover and sugarcane top silage after 60 d of fermentation

Items	OM	CP	EE	NDF	ADF	ADL

	--------------------------------------------------------------------- % of DM ------------------------------------------------------------------------					
Corn stover						
Control	91.98±5.38	6.44±0.59	1.28±0.27	64.69±0.62^a^	36.11±0.28^a^	2.95±0.35^ab^
LAB	91.01±0.40	5.99±0.10	1.61±0.33	64.54±0.54^a^	36.09±0.16^a^	3.26±0.98^a^
Cellulase	88.84±3.30	6.57±0.45	1.33±0.17	59.67±1.04^b^	33.53±0.59^b^	2.05±0.41^b^
LAB+cellulase	88.14±4.52	6.53±0.94	1.42±0.34	60.49±0.79^b^	33.29±0.83^b^	2.27±0.25^ab^
Sugarcane top						
Control	92.76±2.05	6.21±0.83	1.63±0.56	77.89±1.58^a^	46.22±0.97^a^	4.05±0.53^a^
LAB	94.70±1.22	6.00±0.09	1.95±0.15	76.16±0.82^ab^	44.80±1.67^ab^	4.29±0.24^a^
Cellulase	93.25±0.66	6.28±0.52	1.83±0.62	74.97±0.34^b^	43.34±1.27^b^	4.61±0.32^a^
LAB+cellulase	93.90±0.35	6.05±0.37	1.86±0.28	74.79±1.78^b^	44.05±0.21^ab^	4.22±0.12^a^
SEM	1.69	0.33	0.22	0.58	0.52	0.27
Forage means						
Corn stover	89.99±3.74^b^	6.38±0.57	1.41±0.27^b^	62.35±2.48^b^	34.75±1.48^b^	2.63±0.71^b^
Sugarcane top	93.65±1.31^a^	6.14±0.46	1.82±0.40^a^	75.95±1.68^a^	44.60±1.49^a^	4.29±0.36^a^
Additive means						
Control	92.37±3.66	6.32±0.65	1.45±0.44	71.29±7.31^a^	41.16±5.57^a^	3.50±0.72
LAB	92.85±2.34	6.00±0.09	1.78±0.30	70.35±6.39^a^	40.44±4.88^a^	3.77±0.85
Cellulase	91.04±3.22	6.42±0.46	1.58±0.49	67.32±8.41^b^	38.43±5.45^b^	3.33±1.44
LAB+cellulase	91.02±4.27	6.29±0.69	1.64±0.37	67.64±7.93^b^	38.67±5.92^b^	3.24±1.08
Significance of main effects and interactions						
Crops (C)	0.0073	0.3059	0.0164	<0.0001	<0.0001	<0.0001
Additives (A)	0.6179	0.6037	0.5185	<0.0001	0.0001	0.2580
C×A	0.5234	0.8975	0.9777	0.0445	0.2932	0.0344

Data are means of three silage samples.

OM, organic matter; CP, crude protein; EE, ether extract; NDF, neutral detergent fiber; ADF, acid detergent fiber; ADL, acid detergent lignin; DM, dry matter; LAB, lactic acid bacteria inoculant FG; Cellulase, *Acremonium* cellulase; SEM, standard error of the mean.

a,b Means±standard deviation within columns with different superscript letters differ significantly from each other (p<0.05).

**Table 5:** Fermentation quality of corn stover and sugarcane top silage after 60 d of fermentation

Items	DM %	pH	Lactic acid	Acetic acid	Propionic acid	Butyric acid	Ammonia-N (g/kg of DM)

			-------------------------------------- % of DM -------------------------------------				
Corn stover							
Control	31.69±3.05	3.93±0.13^a^	5.00±0.91^a^	1.19±0.21^a^	0.04±0.03^a^	0.01±0.01^b^	0.68±0.14
LAB	31.07±1.78	3.95±0.22^a^	5.14±1.56^a^	1.06±0.16^a^	0.06±0.03^a^	0.03±0.04^b^	0.59±0.09
Cellulase	31.02±1.29	3.95±0.07^a^	4.87±0.74^a^	1.17±0.12^a^	0.09±0.05^a^	0.12±0.05^a^	0.77±0.15
LAB+cellulase	33.19±2.00	3.97±0.12^a^	4.14±0.62^a^	1.10±0.11^a^	0.02±0.01^a^	0.11±0.03^a^	0.76±0.14
Sugarcane top							
Control	27.33±2.67	4.71±0.08^a^	0.61±0.30^b^	1.58±0.14^a^	0.10±0.02^a^	2.49±0.79^a^	1.11±0.11
LAB	27.71±0.04	3.86±0.08^b^	4.82±0.41^a^	0.77±0.17^c^	0.05±0.01^b^	0.46±0.23^b^	0.67±0.07
Cellulase	25.51±0.41	4.09±0.32^b^	3.72±1.57^a^	1.43±0.15^ab^	0.09±0.04^ab^	0.81±0.50^b^	0.83±0.10
LAB+cellulase	27.73±1.20	4.06±0.05^b^	3.30±0.42^a^	1.17±0.22^b^	0.06±0.01^ab^	0.96±0.23^b^	0.73±0.07
SEM	1.06	0.09	0.54	0.10	0.02	0.20	0.06
Crop means							
Corn stover	31.74±2.03^a^	3.95±0.12^b^	4.79±0.96^a^	1.13±0.14	0.05±0.04	0.07±0.06^b^	0.70±0.13
Sugarcane top	27.07±1.58^b^	4.18±0.37^a^	3.11±1.77^b^	1.24±0.35	0.08±0.03	1.18±0.92^a^	0.84±0.19
Additive means							
Control	29.51±3.50	4.32±0.44^a^	2.80±0.48^c^	1.39±0.26^a^	0.07±0.04^ab^	1.25±0.45^a^	0.90±0.26
LAB	29.39±2.16	3.91±0.16^b^	4.98±1.03^a^	0.92±0.21^c^	0.05±0.02^ab^	0.24±0.28^b^	0.63±0.08
Cellulase	28.27±3.14	4.02±0.22^b^	4.30±1.26^ab^	1.30±0.19^ab^	0.09±0.04^a^	0.46±0.12^b^	0.80±0.12
LAB+cellulase	30.46±3.33	4.01±0.10^b^	3.72±0.66^bc^	1.14±0.16^b^	0.04±0.03^b^	0.54±0.49^b^	0.75±0.10
Significance of main effects and interactions							
Crops (C)	<0.0001	0.0027	0.0005	0.1258	0.0664	<0.0001	0.0088
Additives (A)	0.2679	0.0024	0.0073	0.0008	0.0434	0.0008	0.0071
C×A	0.7099	0.0012	0.0072	0.0160	0.2706	0.0005	0.0111

Data are means of three silage samples.

DM, dry matter; LAB, lactic acid bacteria inoculant FG; Cellulase, *Acremonium* cellulase; SEM, standard error of the mean.

a–c Means±standard deviation within columns with different superscript letters differ significantly from each other (p<0.05).

**Table 6:** Microbial population of corn stover and sugarcane top silage after 60 d of fermentation

Item	Lactic acid bacteria	Aerobic bacteria	Coliform bacteria	Yeasts	Molds

	------------------------------------------------------------- Log_10_ cfu /g of FM -----------------------------------------------------------------				
Corn stover					
Control	6.31±0.72^a^	5.02±0.74^a^	ND	5.44±0.58^a^	ND
LAB	6.41±0.40^a^	4.33±0.47^a^	ND	5.57±0.20^a^	ND
Cellulase	6.43±0.43^a^	5.31±0.87^a^	ND	5.30±0.42^a^	ND
LAB+cellulase	6.52±0.20^a^	4.90±0.52^a^	ND	5.54±0.24^a^	ND
Sugarcane top					
Control	4.33±0.39^d^	5.48±0.33^a^	ND	4.23±0.56^a^	ND
LAB	7.22±0.26^a^	3.08±0.06^c^	ND	3.61±0.21^b^	ND
Cellulase	5.48±0.35^c^	4.73±0.08^b^	ND	4.60±0.23^a^	ND
LAB+cellulase	6.38±0.46^b^	3.43±0.25^c^	ND	3.00±0.13^b^	ND
SEM	0.25	0.29	-	0.21	-
Crop means					
Corn stover	6.42±0.41^a^	5.32±1.06^a^	-	5.46±0.35^a^	-
Sugarcane top	5.85±1.17^b^	4.18±1.03^b^	-	4.14±0.54^b^	-
Additive means			-		-
Control	5.32±0.39^c^	6.12±0.33^a^	-	4.83±0.56^a^	-
LAB	6.81±0.26^a^	3.70±0.06^c^	-	4.59±0.21^ab^	-
Cellulase	5.96±0.35^b^	5.02±0.08^b^	-	4.95±0.23^a^	-
LAB+cellulase	6.45±0.46^c^	4.17±0.25^c^	-	4.27±0.13^b^	-
Significance of main effects and interactions					
Crops (C)	0.0052	0.0030	-	<0.0001	-
Additives (A)	<0.0001	0.0002	-	0.0213	-
C×A	0.0003	0.0174	-	0.0021	-

Data are means of three silage samples.

cfu, colony-forming unit; FM, fresh matter; ND, not detected; LAB, lactic acid bacteria inoculant FG; Cellulase, *Acremonium* cellulase; SEM, standard error of the mean; - means the value is zero.

a–d Means±standard deviation within columns with different superscript letters differ significantly from each other (p<0.05).
